# The stories the data can tell: The tenuous link between evidence and theory

**DOI:** 10.1111/medu.70298

**Published:** 2026-08-22

**Authors:** Geoff Norman

**Affiliations:** ^1^ McMaster University Dundas Ontario Canada

## Abstract

Despite years of research there is little evidence that education on biases reduces them. The problem might not lie with the data, but the interpretation. #MedEd

Although we often hear that data speak for themselves, their voices can be soft and sly. (Fred Mosteller)

Science is a special kind of story telling with no right or wrong answers. Just better and better stories. (Mary Budd Rowe)

In this issue of *Medical Education*, Al Essa et al^1^ report on another in their series of elegant experiments designed to elucidate components of the nature of clinical reasoning. Their particular interest is ‘premature closure’, which, as they indicate, has been viewed as a major source of diagnostic errors.

Premature closure is generally viewed as a cognitive bias that leads the clinician to terminate an encounter before gathering the critical data needed to arrive at the correct diagnosis. Their results neatly confirm this hypothesis. The experimental intervention is the salient distracting feature (SDF)—a feature of an alternative diagnosis that is also related to the correct diagnosis. In the study, the SDF was introduced into the case description early, late or not at all. Students working up the case with no SDF had an accuracy of 27%. When the SDF appeared late in the protocol, it dropped to 17%, and when it appeared early, overall accuracy was only 11%. Not surprisingly, percent selecting the distracting diagnosis were almost the inverse—13% when there was no SDF, 30% when it was at the end and 33% when it was at the beginning. Additional data showed that the presence of the SDF resulted in decreased processing time and decreased time per word.

The SDF was persuasive. Students who saw protocols with the SDF were almost twice as likely to conclude for the alternative diagnosis as the correct diagnosis. Moreover, the earlier the SDF was presented the more likely the judgement in favour of the alternative and, perhaps critically, the less time was needed to arrive at this incorrect conclusion.

The authors'^1^ explanation for the results is that ‘One plausible interpretation is that participants accepted an initially plausible but incorrect diagnostic hypothesis and invested less effort in processing subsequently presented information, a pattern consistent with premature closure’. However, as the discussion ensues, it becomes less clear exactly what is going on. The normative view espoused by researchers working in the ‘heuristics and biases’ paradigm would be that the student clinician has a predilection to closing prematurely. An extreme position, taken by Kahneman,^2^ is that such biases are a central aspect of human information processing. We are uniquely vulnerable to various distortions (biases) in thinking as a consequence of our use of cognitive heuristics or shortcuts, which, in turn, are a consequence of our limited working memory. Moreover, these biases cannot be reduced or eliminated by education or experience. As Kahneman^2^ states in his popular book, *Thinking Fast and Slow*:

What can be done about biases? … The short answer is that little can be achieved without a considerable investment of effort. As I know from experience, System 1 is not readily educable. (2, p. 417)

Other researchers, particularly in medicine, reject his extreme position and advocate ‘cognitive debiasing strategies’, where students are taught how to recognize the many potential biases^3^, ^4^ so they can detect biases in their reasoning and hence reduce errors. Al Essa et al^1^ reject this view and correctly, I believe, view premature closure not as a precursor of errors, but as a convenient label for a process whereby a wrong diagnosis is reached. They state:

Premature closure is rather a consequence of the confidence that physicians attach to an early but biased diagnostic hypothesis. The biasing information is the culprit, not premature closure.

They have changed the focus from the overall conclusion that participants in the SDF conditions suffered from premature closure to a process interpretation that the presence of the SDF led them to focus their attention on the plausible incorrect diagnosis.

Then a third explanation is offered. It may be a consequence of ‘confirmation bias’ where participants seek data to confirm the initial wrong diagnosis.

The authors then reject the ‘cognitive bias’ interpretation altogether:

… lack of knowledge as a basis for susceptibility to bias. Elsewhere, we have demonstrated that effects of bias do not emerge when physicians have sufficient appropriate knowledge of the diseases under scrutiny, in particular knowledge of findings discriminating between look‐alike diseases.

There remains one other possible explanation that was not considered by authors. The presence or absence of an SDF is critical to the experiment. But to the participant, it has no special status. It is simply one more piece of clinical information that must be considered in arriving at a diagnosis. To change diagnostic probabilities in light of additional information is not a cognitive bias. Rather, it is precisely what one would expect of a rational analytical diagnostic engine.

Imagine, for a moment, that we feed the written cases into a Bayesian diagnostic system. We ask it to use the data to compute the conditional probabilities of diagnoses A and B under three conditions—no SDF, SDF early and SDF late, with all the data. It is not thinking; it is simply doing straightforward Bayesian analysis using base rates and conditional probabilities. The presence of the SDF will inevitably increase the probability of the alternative diagnosis, although there will be no difference in computed probability between early and late (the effect of early/late is small—see Tables 3 and 4). The results would mirror the present data (except for the early/late difference). We have confirmed this using AI to model a Bayesian diagnosis on the example cirrhosis case.

To summarise, the experiment was elegant and the results interesting and informative. However, interpretation is complicated and ambiguous. I believe this is not a criticism of this study, but rather represents the state of the ‘heuristics and biases’ research programme in general. Despite a five decade history, biases continue to proliferate, often without supporting evidence.^4^, ^5^ Attempts to demonstrate such basic characteristics as agreement among reviewers of the presence of specific biases have produced negative results.^6^ Further, there is little evidence that instruction in recognition of specific cognitive biases will reduce diagnostic errors.^3^

## Data Availability

Data sharing not applicable to this article as no datasets were generated or analysed during the current study.
